# The impact of remote health education for primary home caregivers based on the WeChat platform on home-based rehabilitation outcomes in elderly patients with cerebral infarction

**DOI:** 10.3389/fneur.2026.1786522

**Published:** 2026-03-19

**Authors:** Ruilin Li, Haiyan Gu, Wenzhu Xu, Yijia Sun, Haoyu Wang

**Affiliations:** Department of Critical Care Rehabilitation, Ningbo Rehabilitation Hospital, Ningbo, Zhejiang, China

**Keywords:** cerebral infarction, primary home caregiver, rehabilitation, satisfaction, WeChat

## Abstract

**Objective:**

To explore the impact of remote health education for the primary home caregivers of elderly patients with cerebral infarction via the WeChat platform on patient rehabilitation outcomes.

**Methods:**

A total of 120 elderly patients with cerebral infarction admitted to Ningbo Rehabilitation Hospital from October 2024 to October 2025 and meeting the inclusion criteria were selected as the study subjects. Patients were randomly divided into a control group and an observation group using a random number table. Remote health education for the primary home caregivers of patients in the control group was conducted via telephone follow-up.

**Results:**

The dietary adherence, medication adherence, lifestyle modification adherence, and limb function training adherence in the observation group were significantly higher than those in the control group (*p* < 0.05). The recovery of motor function, activities of daily living, and neurological function in the observation group was significantly better than that in the control group. The quality of life score in the observation group was significantly higher, while anxiety and depression scores were significantly lower than those in the control group (*p* < 0.05). The overall effective rate and satisfaction with home rehabilitation were also significantly higher than those in the control group (*p* < 0.05).

**Conclusion:**

Remote health education for elderly patients with cerebral infarction based on the WeChat platform significantly improves patient rehabilitation adherence, quality of life, and alleviates negative emotions, markedly enhancing the home-based rehabilitation outcomes for elderly cerebral infarction patients.

## Introduction

Cerebral infarction, also known as stroke, is an acute condition frequently occurring in middle-aged and elderly populations and can even threaten patients’ lives. The “China Stroke Declaration” issued by the former National Health and Family Planning Commission in 2018 indicated that the incidence of cerebral infarction in China continues to rise and has become the leading cause of death among Chinese residents (1). Despite continuous advancements in clinical treatment techniques, a significant proportion of surviving patients still experience varying degrees of disability (2). Limited by China’s eldercare models and economic development level, most elderly patients with cerebral infarction choose to continue rehabilitation treatment at home after their condition stabilizes. However, due to the lack of professional knowledge among the primary home caregivers of these patients, the rehabilitation outcomes are often suboptimal (3). As the caregivers who spend the most time with the patients, the primary home caregivers’ mastery of knowledge regarding cerebral infarction and rehabilitation is closely related to the recovery of these patients (4). Currently, there are numerous methods for implementing health education during the patient rehabilitation process, such as telephone follow-ups, email support, and establishing patient clubs for post-discharge health education (5). With the development of internet technology and online platforms, WeChat has become one of the communication methods with extensive coverage and high frequency of use. The emergence of the WeChat platform enables the primary home caregivers of cerebral infarction patients to acquire accurate and professional nursing knowledge without leaving home, offering advantages of easy implementation and high feasibility (6, 7). This study conducted remote health education for the primary home caregivers of cerebral infarction patients via the WeChat platform, which can significantly improve the rehabilitation outcomes of elderly cerebral infarction patients and is worthy of clinical promotion. The details are reported as follows.

## Methods

### General information

A total of 120 elderly patients with cerebral infarction admitted to Ningbo Rehabilitation Hospital from October 2024 to October 2025 and meeting the inclusion criteria were selected as the study subjects. Inclusion criteria: Age ≥60 years; Clinical diagnosis met the diagnostic criteria for cerebral infarction outlined in the “Chinese Classification of Cerebrovascular Diseases 2015” (8); No concurrent major organ dysfunction; No concurrent diseases such as schizophrenia or intellectual disability; capable of independently completing relevant questionnaires; Patients opted for home-based rehabilitation after treatment; The primary home caregiver of the patient could skillfully use WeChat for effective communication; Both the patient and family members were informed of the study’s purpose and significance. Using a computer-generated random sequence, a researcher not involved in recruitment or intervention allocated group information into sequentially numbered, sealed, opaque envelopes for allocation concealment. All 120 patients were enrolled and randomly divided into a control group and an observation group, with 60 patients in each group. In the control group, there were 34 males and 26 females, aged 60–82 years (mean age 70.05 ± 6.48 years). In the observation group, there were 32 males and 28 females, aged 61–83 years (mean age 71.93 ± 5.63 years). Comparisons of baseline data, including age, gender, and severity of cerebral infarction, between the two groups showed no statistically significant differences (*p* > 0.05), indicating comparability. Due to the differences in intervention forms, blinding was not implemented for the patients and their primary caregivers. However, all outcome assessments were performed by dedicated assessors unaware of the patients’ group allocation. This study was approved by the Ethics Committee of Ningbo Rehabilitation Hospital (Approval No. 2024-021) and informed consent was provided by all participants.

### Research methods

The primary home caregivers of patients in the control group received routine discharge guidance via telephone follow-up, including medication usage methods, rehabilitation training methods, and dietary structure adjustment for the patients. The home-based rehabilitation team for the observation group consisted of six experienced medical staff (two neurologists, two rehabilitation therapists, and two nurses), each responsible for 10 patients. The intervention lasted for 4 weeks, with 2–3 training sessions per week via WeChat group chats, each session lasting 30–45 min. Building upon the discharge guidance for the control group, knowledge about cerebral infarction based on the “Stroke Health Education Guidelines” (9) was disseminated to the primary home caregivers of the observation group via WeChat group chats, and medication use and rehabilitation training were guided through video calls. The rehabilitation training content was individualized according to each patient’s functional level and mainly included: (1) passive and active range of motion exercises for affected limbs; (2) bed mobility and transfer training; (3) sitting and standing balance training; (4) activities of daily living training (dressing, eating, grooming, etc.); and (5) fall prevention strategy guidance. Relevant surveys and statistics were conducted during the patients’ follow-up visit 4 weeks after discharge.

### Observation indicators

#### Rehabilitation process adherence in both groups

A self-made compliance questionnaire for cerebral infarction patients was used to compare the rehabilitation process adherence between the two groups from four aspects: dietary adherence, medication adherence, lifestyle modification adherence, and limb function training adherence. The total score of this questionnaire is 100 points: <60 points indicates non-compliance; 60–80 points indicates partial compliance; >80 points indicates full compliance. Adherence rate = [(Number of patients with partial compliance + Number of patients with full compliance)/Total number of patients × 100]%. The questionnaire was developed based on a comprehensive literature review and expert consultation, and was piloted to ensure the items were clear and comprehensible.

#### Rehabilitation efficacy in both groups

The Fugl-Meyer Assessment (FMA) scale was used to evaluate the patients’ abilities in unsupported sitting, supported standing, parachute reaction on the unaffected side, parachute reaction on the affected side, standing on the unaffected side, and standing on the affected side. Each item scored 0–2 points, with a total score of 14 points; higher scores indicate better motor function. The Modified Barthel Index (MBI) was used to evaluate the activities of daily living (ADL) of patients in both groups, with a total score of 100 points; higher scores indicate better ADL. The National Institutes of Health Stroke Scale (NIHSS) was used to evaluate the neurological function of patients in both groups, with a total score of 22 points; higher scores indicate worse neurological function.

#### Quality of life in both groups

The Quality of Life (QOL) scale (10) was used to assess patients’ quality of life from aspects such as health status, general information, and daily life, with a total score of 100 points; higher scores indicate better quality of life. The Self-Rating Anxiety Scale (SAS) and Self-Rating Depression Scale (SDS) (11) were used to evaluate the levels of anxiety and depression in both groups, with a total score of 100 points; higher scores indicate more severe anxiety and depression.

#### Home-based rehabilitation efficacy and satisfaction in both groups

Rehabilitation efficacy criteria: Markedly effective: Clinical symptoms disappeared, condition essentially recovered; Effective: Clinical symptoms largely disappeared, condition effectively improved; Ineffective: No significant improvement in condition. A self-made patient home-based rehabilitation satisfaction questionnaire was used to evaluate satisfaction, with a total score of 100 points: <60 points indicates dissatisfaction; 60–80 points indicates basic satisfaction; >80 points indicates very satisfied. Satisfaction rate = [(Number of patients with basic satisfaction + Number of patients with very satisfied)/Total number of patients × 100]%. The questionnaire underwent expert review and pilot testing to ensure its content relevance and comprehensibility.

#### Satisfaction with remote health education and mastery of related knowledge among primary home caregivers in both groups

A self-made satisfaction questionnaire for remote health education among primary home caregivers was used to assess their satisfaction, with a total score of 100 points: <60 points indicates dissatisfaction; 60–80 points indicates basic satisfaction; >80 points indicates very satisfied. Satisfaction rate = [(Number of caregivers with basic satisfaction + Number of caregivers with very satisfied)/Total number of caregivers × 100]%. A questionnaire on cerebral infarction prevention and treatment knowledge was used to assess the mastery of cerebral infarction-related knowledge among the primary home caregivers in both groups, with a total score of 36 points; higher scores indicate better knowledge mastery. The above questionnaires were all developed based on literature review and expert consultation, and were piloted to ensure item clarity and comprehensive content.

### Statistical processing

Statistical analysis was performed using SPSS 23.0 software. Measurement data conforming to a normal distribution are expressed as mean ± standard deviation ( $\bar{x}\pm s$ ), and inter-group comparisons were conducted using the independent samples *t*-test. Measurement data not conforming to a normal distribution are expressed as median (interquartile range) [M (IQR)], and inter-group comparisons were conducted using the Mann–Whitney *U* test. Count data are expressed as number (percentage) [*n* (%)] and analyzed using the *χ*^2^ test. A *p*-value < 0.05 was considered statistically significant.

## Results

### Comparison of patient adherence to home-based rehabilitation therapy

No cases were lost to follow-up during the study; all 120 patients completed the study. The dietary adherence, medication adherence, lifestyle modification adherence, and limb function training adherence in the observation group were higher than those in the control group, with the differences being statistically significant (*p* < 0.05), as shown in Table 1.

**Table 1:** Comparison of patients’ compliance with rehabilitation treatment [*n* (%)].

Group	Number of cases	Diet	Medication	Lifestyle modification	Limb function training
Control group	60	50 (83.33)	56 (93.33)	43 (71.67)	46 (76.67)
Observation group	60	57 (95.00)	60 (100.00)	52 (86.67)	57 (95.00)
$\chi^{2}$	—	4.227	4.138	4.093	8.292
*p*	—	0.040	0.042	0.043	0.004

### Comparison of functional recovery during home rehabilitation

The median FMA score and median MBI score in the observation group were higher than those in the control group, while the median NIHSS score was lower than that in the control group, with statistically significant differences (*p* < 0.05). This indicates that the recovery of motor function, activities of daily living, and neurological function in the observation group were superior to those in the control group, as shown in Table 2.

**Table 2:** Comparison of functional recovery of patients ( $\bar{x}\pm s$ ).

Group	Number of cases	FMA	MBI	NIHSS
Control group	60	12 (11, 13)	73.5 (67.0, 78.0)	7.0 (5.0, 8.0)
Observation group	60	14 (12, 14)	85.0 (77.0, 88.0)	5.0 (4.0, 5.0)
*U*	—	961.5	753.0	450.5
*p*	—	<0.001	<0.001	<0.001

### Comparison of quality of life improvement during home rehabilitation

The QOL, SAS, and SDS scores in the observation group were lower than those in the control group, with statistically significant differences (*p* < 0.05). The QOL score in the observation group was significantly higher than that in the control group, while the SAS and SDS scores were significantly lower (*p* < 0.05). This suggests that patients in the observation group had a higher quality of life and lower levels of anxiety and depression, as shown in Table 3.

**Table 3:** Comparison of patients’ quality of life ( $\bar{x}\pm s$ ).

Group	Number of cases	QOL	SAS	SDS
Control group	60	88.27 ± 3.62	48.70 ± 5.36	51.83 ± 6.15
Observation group	60	93.30 ± 3.43	42.48 ± 4.81	44.42 ± 5.36
*t*	—	7.815	6.683	7.040
*p*	—	<0.001	<0.001	<0.001

### Comparison of home rehabilitation efficacy and satisfaction

The total effective rate of home rehabilitation in the observation group was 96.67%, which was higher than the 86.67% in the control group, with a statistically significant difference (*p* < 0.05), as shown in Table 4. Additionally, patient satisfaction with home rehabilitation was significantly higher in the observation group compared to the control group [58 (96.67%) vs. 52 (86.67%)], with a statistically significant difference (*p* < 0.05).

**Table 4:** Comparison of rehabilitation effect of patients at home [*n* (%)].

Group	Number of cases	Markedly effective	Effective	Ineffective	Total effective rate
Control group	60	37 (61.67)	15 (25.00)	8 (13.33)	52 (86.67)
Observation group	60	42 (70.00)	16 (26.67)	2 (3.33)	58 (96.67)
$\chi^{2}$	—	3.927			
*p*	—	0.048			

### Comparison of satisfaction with remote health education and knowledge mastery among primary home caregivers

The satisfaction of primary home caregivers in the observation group with remote health education was higher than that in the control group [60 (100.00%) vs. 56 (93.33%)]. Their mastery of cerebral infarction-related knowledge was also higher than that in the control group (25.38 ± 3.52 vs. 20.53 ± 2.67), with statistically significant differences (*p* < 0.05).

## Discussion

Cerebral infarction is a common disease in neurology, characterized by acute onset and a prolonged recovery period, imposing significant psychological and economic burdens on patients and their families (12). The focus of treatment and rehabilitation for cerebral infarction lies in the acute phase, with the first 3 months post-onset being the optimal period for rehabilitation (13). However, primary home caregivers often lack basic medical knowledge. A scoping review by Backman et al. (14) on digital health interventions during the hospital-to-home transition period highlighted that platform-based digital interventions between healthcare providers and patients are an important means to improve communication, coordination, and information sharing during this transition. Among the 97 studies included, stroke-related interventions accounted for 11%, confirming the application value of this model in neurological rehabilitation. Through the WeChat platform, primary home caregivers can communicate timely with professional medical staff during the patient’s home rehabilitation. Additionally, guidance can be provided to primary caregivers via voice calls, video chats, image sharing, and text messages, thereby enhancing their caregiving capabilities. A systematic review and meta-analysis by Tchero et al. (15) provided supporting evidence for this. The study, which pooled 12 randomized controlled trials, found that for stroke patients, telerehabilitation was comparable to conventional rehabilitation care in improving activities of daily living (Barthel Index), balance function (Berg Balance Scale), and patient satisfaction, with potential cost-effectiveness advantages. This strongly supports that the remote guidance model represented by WeChat, as a lightweight practice of structured telerehabilitation, can achieve effects comparable to traditional home rehabilitation guidance, serving as an effective way to meet the needs of primary home caregivers for professional medical knowledge. However, it is important to note that Backman’s et al. (14) review also identified “lack of interest,” “time constraints” and “limited technology or internet access” as major barriers. This highlights the need to fully consider caregivers’ willingness, time costs, and the digital divide when promoting WeChat-based interventions. Furthermore, hospitals and medical staff can review chat records with primary home caregivers or patients to identify gaps and improve rehabilitation guidance plans for cerebral infarction patients.

A study by Lu and Bao (16) indicated that implementing continuity of care for rectal cancer patients with colostomy via the WeChat platform significantly improved their quality of life and reduced the risk of complications. Similarly, research by Yang et al. (17) demonstrated that health manager-centered multi-channel health education significantly improved patients’ treatment compliance after discharge and markedly reduced adverse activity behaviors. In this study, professional medical staff provided remote health education to primary home caregivers of elderly cerebral infarction patients through WeChat group chats and video calls, guiding them in home-based rehabilitation. Compared to the control group, which received traditional remote education, the observation group showed significantly higher compliance with home rehabilitation treatment, better recovery across various functions, improved quality of life, and effective control of negative emotions. Additionally, the total effective rate of home rehabilitation in the observation group was significantly higher. This study also found that through remote health education via WeChat, primary home caregivers could repeatedly review and learn from the professional knowledge shared by medical staff in chat records, consolidating their understanding. As a result, they were more receptive to and achieved higher mastery of cerebral infarction-related knowledge.

This study has several limitations. First, the intervention duration was relatively short (4 weeks). While early positive changes in patient compliance, mood, and some functional aspects were observed, it remains unclear whether these positive effects can be sustained in the long term, given that post-cerebral infarction rehabilitation is a prolonged and continuous process. Future studies with longer follow-up periods are needed to verify the durability of these effects. Second, the outcome assessments in this study were primarily based on scale scores. Furthermore, due to the specific nature of the intervention, blinding of the patients and their primary caregivers was not implemented, which may have introduced some degree of measurement bias or social desirability bias. Third, some of the assessment tools used in this study (e.g., the compliance questionnaire, satisfaction questionnaire, and stroke knowledge questionnaire) were self-developed by the researchers. Although they underwent expert review and pilot testing to ensure content validity, formal reliability and validity testing was not conducted. Future studies are recommended to include statistician involvement in the review process and to conduct systematic psychometric validation of these instruments. Fourth, the allocation of six clinical staff members to intervene with 60 patients represents a relatively high resource investment. However, considering the complexity of post-stroke home-based rehabilitation and the potential value of personalized guidance in improving patient outcomes, this staffing model can be considered an exploratory approach. Future research should incorporate health economic evaluations to comprehensively assess its cost-effectiveness. Finally, the participants in this study were from caregiver families proficient in using WeChat. Therefore, the results may not be directly generalizable to elderly populations with lower digital literacy or those without access to smartphones. This indicates a need to explore more diversified support models in the future.

In summary, remote health education for primary home caregivers via the WeChat platform is effective in improving home rehabilitation outcomes for elderly cerebral infarction patients, enhancing their compliance with home rehabilitation treatment, and is worthy of clinical promotion and application.

## Data Availability

The raw data supporting the conclusions of this article will be made available by the authors, without undue reservation.
